# The Effectiveness of Virtual Reality–Based Interventions in Rehabilitation Management of Breast Cancer Survivors: Systematic Review and Meta-analysis

**DOI:** 10.2196/31395

**Published:** 2022-02-28

**Authors:** Xiaofan Bu, Peter H F Ng, Wenjing Xu, Qinqin Cheng, Peter Q Chen, Andy S K Cheng, Xiangyu Liu

**Affiliations:** 1 Nursing Teaching and Research Section Hunan Cancer Hospital/The Affiliated Cancer Hospital of Xiangya School of Medicine Central South University Changsha China; 2 Department of Computing The Hong Kong Polytechnic University Hong Kong China; 3 Xiangya School of Nursing Central South University Changsha China; 4 The Nethersole School of Nursing Faculty of Medicine The Chinese University of Hong Kong Hong Kong China; 5 Department of Rehabilitation Sciences The Hong Kong Polytechnic University Hong Kong China; 6 Department of Health Service Center Hunan Cancer Hospital/The Affiliated Cancer Hospital of Xiangya School of Medicine Central South University Changsha China

**Keywords:** virtual reality, rehabilitation management, symptom, motor function, systematic review, meta-analysis

## Abstract

**Background:**

Breast cancer survivors (BCSs) can present with various physical and psychological symptoms and functional deficits that impact their quality of life. Virtual reality (VR) technology is being used in breast cancer rehabilitation management to improve the emotional, cognitive, and physical well-being of BCSs.

**Objective:**

This systematic review aimed to examine the effectiveness of VR-based interventions on health-related outcomes in BCSs. A meta-analysis was conducted to evaluate the effectiveness of VR-based interventions in the rehabilitation management of BCSs.

**Methods:**

A systematic search was conducted on PubMed, Web of Science, EMBASE, CINAHL with Full Text, the Cochrane Central Register of Controlled Trials, CNKI, WanFang, VIP, and CBM, from inception to May 25, 2021. The inclusion criteria of the selected studies were as follows: (1) adults diagnosed with breast cancer; (2) any type of VR-based interventions (immersive and nonimmersive virtual environment); (3) comparison of traditional rehabilitation methods; (4) outcomes including pain, depression, anxiety, fatigue, cognitive function, shoulder range of motion (ROM), hand grip strength, lymphedema, cybersickness symptoms, fear of movement, bleeding, effusion, and flap necrosis, both during and after treatment; and (5) randomized controlled trials (RCTs), case-controlled trials, and quasi-experimental studies. The Cochrane Collaboration Tool was used to evaluate the risk of bias. Review Manager version 5.3 (Cochrane Collaboration) was used to conduct the meta-analysis. The mean difference (MD) and SDs with 95% CIs were used to calculate continuous variables.

**Results:**

Twelve articles were included in this systematic review, of which 10 contributed information to the meta-analysis. A total of 604 participants were analyzed. The statistical analysis showed significant results for flexion (standard mean difference [SMD] 1.79; 95% CI 0.55 to 3.03; *P*=.005), extension (SMD 1.54; 95% CI 0.83 to 2.25; *P*<.001), abduction (MD 17.53; 95% CI 14.33 to 20.72; *P*<.001), adduction (MD 15.98; 95% CI 14.02 to 17.94; *P*<.001), internal rotation (MD 7.12; 95% CI 5.54 to 8.70; *P*<.001), external rotation (SMD 0.96; 95% CI 0.62 to 1.29; *P*<.001), anxiety (MD −6.47; 95% CI −7.21 to −5.73; *P*<.001), depression (MD −4.27; 95% CI −4.64 to −3.91; *P*<.001), pain (MD −1.32; 95% CI −2.56 to −0.09; *P*=.04), and cognitive function (MD 8.80; 95% CI 8.24 to 9.36; *P*<.001). The meta-analysis indicated little to no difference in hand grip strength (MD 1.96; 95% CI –0.93 to 4.85; *P*=.18).

**Conclusions:**

Findings of this review noted a weak but consistent positive association between VR-based interventions and outcomes. However, these results must be interpreted with caution due to the limited number of controlled trials analyzed, small sample sizes, and poor methodological quality. Well‐designed, large, high‐quality trials may have a significant impact on our confidence in the results. Future studies should identify specific aspects that improve the clinical impact of VR-based interventions on major outcomes in BCSs in the clinical setting.

**Trial Registration:**

PROSPERO International Prospective Register of Systematic Reviews CRD42021250727; https://tinyurl.com/2p89rmnk

## Introduction

Female breast cancer has surpassed lung cancer as the most commonly diagnosed cancer worldwide, with an estimated 2.3 million new cases in 2020 [1]. The 5-year relative survival rate for individuals with breast cancer is 82% [2]. An increasing number of patients with breast cancer have prolonged life following treatment; however, they can suffer from numerous physical and psychological symptoms (ie, pain, fatigue, depressive symptoms, anxiety, lymphedema), functional deficits (ie, cognitive impairment, reduced shoulder range of motion [ROM]), and complications (ie, bleeding, effusion, flap necrosis) during and after treatment, which can greatly affect their quality of life [3-5].

While chemotherapy improves the survival rate of patients with cancer, the potential adverse effects of chemotherapy limit the dose and treatment continuation. To some extent, adverse effects can aggravate patients’ emotional distress. Emotional distress mainly includes fatigue, pain, anxiety, and depression, which is commonplace in cancer populations [6]. Distress was designated as the sixth vital sign in 2005 in Canada [7] associated with a reduction in overall quality of life among patients with cancer [8]. Cancer-related fatigue is also distressing, persistent, and related to a subjective sense of physical, emotional, or cognitive tiredness or exhaustion related to cancer or cancer treatment, which is not proportional to recent activity and interferes with general functioning [9]. The level of cancer-related fatigue peaks during breast cancer therapy, and the prevalence of chronic fatigue increases after treatment [10]. The prevalence rates of severe fatigue range from 7% to 52%, with a pooled prevalence of 26.9%. Risk factors of fatigue were higher disease stages, chemotherapy, and receiving the combination of surgery, radiotherapy, and chemotherapy, both with and without hormone therapy [11]. Patients with anemia are prone to fatigue owning to the reduced hemoglobin level after chemotherapy [12]. The prevalence of depression symptoms varies from 9.4% to 66.1%, whereas that of anxiety ranges from 17.9% to 33.3% [13]. Age, place of residence, marital status, educational level, religion, stage of cancer, and current activity burden of symptoms were found to be factors associated with the risk of anxiety and depression [8]. Anxiety did not show greater prevalence among women with early stage breast cancer in comparison to the general female population [13]. Breast cancer survivors (BCSs) have been shown to have an increased risk of depression 1 year after diagnosis, which decreases over the ensuing years [13]. Early interventions can improve treatment tolerance, which could be crucial to increase the chances of recovery [14]. Virtual reality (VR) is the use of computer technology to create an interactive 3D world by visual, audio, and touch simulation, where an individual has a sense of spatial presence. VR could be a promising strategy to improve chemotherapy tolerance by distraction. VR can include immersive or nonimmersive systems. With full immersive systems, the patient is enveloped in a computer-generated virtual world by using a head-mounted display and has opportunities to interact with and control the virtual environment (eg, relaxing landscapes, deep sea diving, the weather, plants/trees, or flowers) [15-17]. With nonimmersive systems, the patient is connected to the virtual world (eg, emotional parks and walk through nature) by an external monitor but can still communicate with the real world [18]. Nonimmersive system is intuitive and easy to use [18].

Pain related to cancer is a distressing experience, with sensory, emotional, cognitive, and social components [19,20]. The prevalence of persistent postsurgical pain in BCSs ranges from 2% to 78% [21]. Fear of movement further increases the risk of decline in upper limb function in BCSs [22]. However, avoiding movements that are likely to induce pain may aggravate upper limb dysfunction. VR exposure can target cognitive and affective pain pathways [23] and can decrease pain intensity, distress, and anxiety by altering how pain signals are processed in the central nervous system [23]. This is achieved by a series of mechanisms, including attentional distraction, conditioning of VR imagery, and reduced pain [23].

BCSs are at a lifelong risk for the development of breast cancer–related lymphedema (BCRL) [24], which has an incidence of 21.4% [25]. There is strong evidence that higher BMI, larger number of dissected nodes, certain chemotherapy agents (eg, taxane-based regimen), the extent of surgery (eg, total mastectomy), larger irradiation field, and sedentary lifestyles are associated with BCRL [25-27]. Disruption of the lymphatic system after surgery or radiation treatment results in the accumulation of lymph fluid causing BCRL [28]. BCRL is a chronic, potentially debilitating condition that involves progressive swelling, limited ROM, and feelings of pain and numbness, and requires lifelong symptom management [24]. Resistance exercise ameliorates symptoms in patients with established lymphedema [29]. VR-based rehabilitation systems (eg, Xbox 360 Kinect games, the BrightArm Duo Rehabilitation System) have been identified to be effective for patients with weak arms and diminished grasping ability [22,30]. These systems use VR to engage the patient in upper body bimanual exercises. Moreover, the BrightArm Duo Rehabilitation System simultaneously provides cognitive training and affective relief via custom integrative rehabilitation games. Cancer-related cognitive impairment is characterized as deficits in areas of cognition, including memory, attention, information processing speed, and executive function [31,32]. Between 15% and 50% of individuals with breast cancer who receive chemotherapy experience persisting cognitive impairment [33], often referred to as “chemobrain” [34]. The duration of symptoms may extend for years after the completion of treatment [31]. The rapid development of VR promotes the combination of functional rehabilitation and cognitive exercises at a higher level, where patients can receive bimanual and cognitive exercises simultaneously.

However, it is unclear whether VR-based interventions could promote the rehabilitation management of BCSs. Additionally, until now, no systematic reviews or meta-analyses have investigated the association between VR and rehabilitation management of BCSs. Therefore, in this systematic review and meta-analysis, we will qualitatively and quantitatively examine the effects of VR-based interventions on BCSs.

## Methods

### Overview and Registration

This systematic review conforms to the Preferred Reporting Items for Systematic Reviews and Meta-Analyses (PRISMA) statement [35] and was registered in advance in the international Prospective Register of Systematic Reviews (PROSPERO) database (registration number CRD42021250727).

### Search Strategy

The literature search was conducted on PubMed, Web of Science, EMBASE, CINAHL with Full Text, the Cochrane Central Register of Controlled Trials, CNKI, WanFang, VIP, and CBM, from inception to May 25, 2021. The search terms were chosen to be inclusive of VR (eg, “virtual reality”, “VR”, “virtual environment”) and breast cancer (eg, “breast neoplasm” OR “breast tumors”). Medical Subject Headings (MeSH) and Embase Subject Headings terms were used. See Multimedia Appendix 1 for the specific search strategy adapted for each database. Details of the search strings of the PubMed database are displayed in Table 1. Searches were limited to English and Chinese language sources.

**Table 1:** Search strategy in PubMed.

Strategy	Search string
1	“Virtual Reality”[Mesh] OR VR OR “virtual reality” OR “virtual environment” OR “head-mounted display” OR “virtual reality goggle”
2	“Breast Neoplasms”[Mesh] OR “breast neoplasms” OR “breast neoplasm” OR “breast tumors” OR “breast tumor” OR “breast cancer” OR “mammary cancer” OR “mammary cancers” OR “Breast Malignant Neoplasm” OR “Breast Malignant Neoplasms” OR “Malignant Tumor of Breast” OR “Breast Malignant Tumor” OR “Breast Malignant Tumors” OR “Cancer of Breast” OR “Cancer of the Breast” OR “Mammary Carcinoma” OR “Human Mammary Carcinomas” OR “Human Mammary Carcinoma” OR “Mammary Neoplasms” OR “Human Mammary Neoplasm” OR “Human Mammary Neoplasms” OR “Mammary Neoplasm” OR “Breast Carcinoma” OR “Breast Carcinomas”
3	#1 AND #2

### Selection Criteria

The Population, Intervention, Comparison, Outcomes, and Study design (PICOS) model was used to establish the article inclusion criteria:

- *Population:* adults diagnosed with breast cancer;
- *Intervention:* any type of VR-based interventions (immersive and nonimmersive virtual environment);
- *Comparison:* traditional rehabilitation methods (including interventions under the guidance of medical staff or watching videos) or nonintervention;
- *Outcomes:* outcomes specifically related to rehabilitation management, such as pain, depression, anxiety, fatigue, cognitive function, shoulder ROM, hand grip strength, lymphedema, cybersickness symptoms, fear of movement, bleeding, effusion, and flap necrosis after surgery; and
- *Study design:* randomized controlled trials (RCTs), case-controlled trials, and quasi-experimental studies.

Studies were excluded if they (1) did not specify the type of cancer; (2) described the technologies only; (3) were conference papers, workshop papers, literature reviews, posters, comments, letters, study protocols, or proceedings papers.

### Selection Process

Records from searches were imported into an EndNote library (EndNote X9.1) and duplicate studies were removed. The remaining records were transferred into an Excel spreadsheet (Microsoft). Screening was conducted by 2 independent reviewers (XB and WX) who assessed the article titles, abstracts, and full texts. Articles that did not meet the established inclusion criteria were excluded. Any disagreements between the 2 reviewers were resolved by discussion or in consultation with other investigators (QC, AC, and XL).

### Data Extraction

Data extraction was performed independently by 2 reviewers (XB and WX) using a predesigned standardized form in Word (Microsoft). Any discrepancies between the 2 reviewers were resolved by discussion with other reviewers (QC, AC, and XL), who acted as arbiters where necessary. We removed duplicate data published in different manuscripts. Additionally, the authors of the included trials were contacted to obtain any unclear or missing data. Data extraction included study characteristics (the first author, study design, and study region), participant characteristics (sample size, age), intervention details (characteristics of interventions, duration), patient-important outcomes, measuring instrument, and main results.

### Risk of Bias Assessment

Two reviewers (XB and WX) independently assessed the methodological quality of all included trials. The Cochrane risk-of-bias tool was used to assess the quality of included RCTs [36]. The Cochrane risk-of-bias tool includes 6 domains of bias: selection bias, performance bias, detection bias, attrition bias, reporting bias, and other bias. After assessing the risk of bias of each study, the studies were categorized as “low risk,” “high risk,” or “unclear risk.” The Risk Of Bias In Non-randomized Studies of Interventions (ROBINS-I) was used to assess the quality of the included non-RCTs, covering 7 distinct domains: bias due to confounding, selection bias, bias in measurement classification of interventions, bias due to deviations from intended interventions, bias due to missing data, bias in the measurement of outcomes, and bias in the selection of the reported result [37]. The ROBINS-I is a new tool for evaluating the risk of bias in estimates of the comparative effectiveness (harm or benefit) of interventions from studies that do not use randomization to allocate units (individuals or clusters of individuals) to comparison groups [37]. After assessing the risk of bias of each study, the studies were categorized as “low risk of bias,” “moderate risk of bias,” “serious risk of bias,” “critical risk of bias,” or “no information.” In case of doubt, the final decision was determined through discussion or consultation with other reviewers (QC, AC, and XL).

### Meta-analysis

The meta-analysis compared VR interventions with other interventions or nonintervention. The studies were divided into subgroups based on the measuring instrument that was used in the study. If more than 1 instrument was used in the same study, we included the study in more than 1 subgroup. The differences in the effect size between the groups were analyzed in terms of the standardized mean difference (SMD). Review Manager version 5.3 (Cochrane Collaboration) was used to conduct a meta-analysis. The mean difference (MD) and SDs with 95% CIs were used to calculate continuous variables. Initially, a fixed-effect model was used in the data analysis. An *I*^2^ value over 0.5 was considered to represent substantial heterogeneity and a random-effect model was used [38]. Subgroup analyses were not possible due to the lack of patient-level data. All *P* values were 2 sided.

## Results

### Search Output

**Figure 1:**
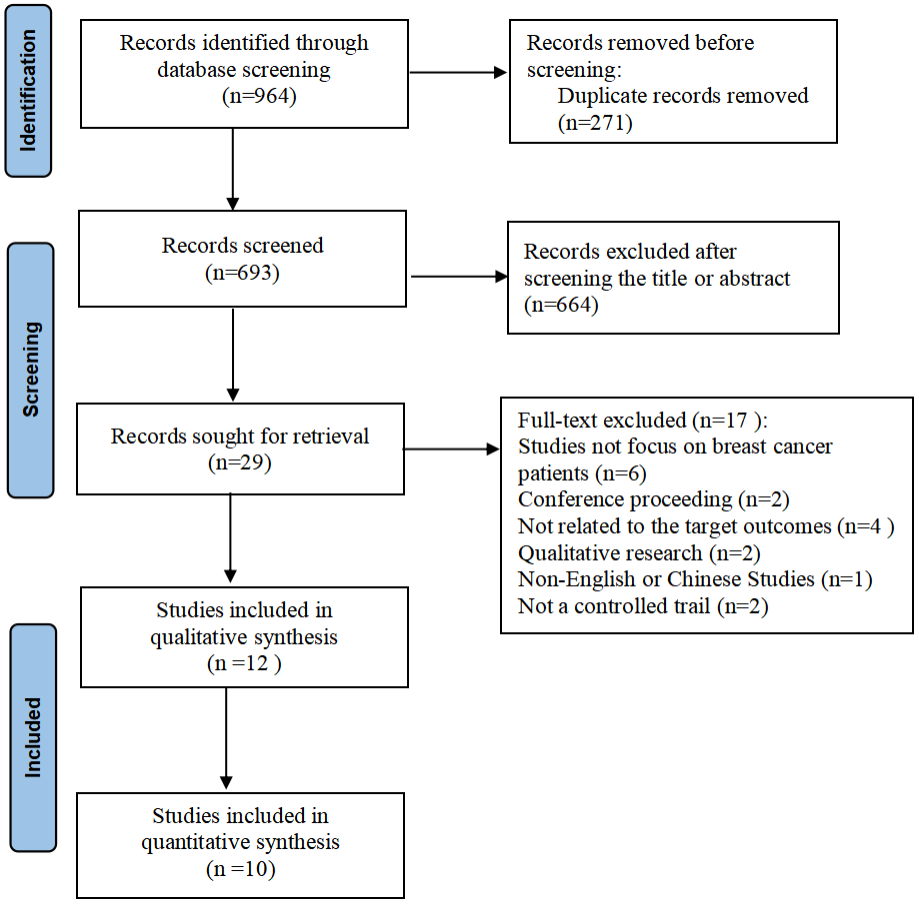
PRISMA (Preferred Reporting Items for Systematic Reviews and Meta-Analyses) flowchart.

A total of 964 potentially relevant articles were initially identified from the 9 databases; 271 articles were removed due to duplication, and the remaining 693 studies were screened. We excluded 664 articles due to insufficient relevance based on the title and abstract. The characteristics of the excluded studies are shown in the PRISMA diagram (Figure 1). Twelve studies were included in the systematic review, 10 of which were further included in the meta-analysis.

### Characteristics of the Included Studies

The characteristics of the 12 studies are shown in Table 2. Three studies were from the United States, 3 from China, and 1 each from Turkey, Italy, Egypt, France, Australia, and Jordan. The included studies were published between 2003 and 2021 [14-17,23,30,39-44]. Of the 12 studies, 6 were RCTs [15,16,23,41-43], 2 were quasi-experimental design studies [39,40], 1 was an externally controlled trial [14], and 3 [17,30,44] were pre–posttest study designs with a single arm. The number of participants ranged from 6 to 80. All participants were adult patients with breast cancer or BCSs. VR-based interventions included both immersive and nonimmersive formats. The intervention duration varied from 15 minutes to 10 months. All studies examined the effects of VR-based interventions on health-related outcomes, including shoulder ROM, hand grip strength, anxiety, depression, pain reduction, cognitive function, fatigue, incidence of complications, cybersickness symptoms, and fear of movement.

**Table 2:** Characteristics of the 12 studies.

Author [reference], country	Study design	Study sample	Intervention methods	Intervention duration	Outcome/instrument	Main results
Feyzioğlu et al [22], Turkey	RCT^a^	Forty women with breast cancer were randomly assigned to the experimental group (use of Xbox 360 Kinect–based VR^b^ training) and the control group (standardized physical therapy group).	Xbox 360 Kinect–based VR training: using Kinect Sports I (darts, bowling, boxing, beach volleyball, table tennis) and Fruit Ninja.	A total of 6 weeks of exercising with the Kinect (duration of 35 minutes/day for 2 days per week).	VAS^c^, ROM^d^, arm strength, DASH^e^ questionnaire, TKS^f^	Significant changes in pain, ROM, muscle strength, grip strength, functionality, and TKS scores after the treatment (*P*<.05).
Chirico et al [14], Italy	Externally controlled trial	Patients were randomly assigned to the VR or music group (MT) and were compared with a nonconcurrently recruited control group. Thirty patients were included in the VR intervention group, 30 patients in the MT intervention group, and 34 patients constituted the control group.	Vuzix Wrap 1200VR head-mounted glasses with the Second Life platform was used to explore an island, walk through a forest, observe different animals, climb a mountain, and swim in the sea.	Patients used the equipment for 20 minutes during chemotherapy.	SAI^g^, SV-POMS^h^, VRSQ^i^	VR and MT are useful interventions for alleviating anxiety and for improving mood states in patients with breast cancer during chemotherapy (*P*<.05). VR seems more effective than MT in relieving anxiety, depression, and fatigue.
Atef et al [39], Egypt	Quasi-randomized clinical trial	Fifteen participants were assigned to the experimental group (use of Nintendo Wii) and 15 to the control group (proprioceptive neuromuscular facilitation).	Nintendo Wii game: tennis, triceps extension, and rhythmic boxing.	The duration of the VR-based therapy sessions included 30 minutes of training over a period of 4 weeks, with 2 sessions every week.	Circumferential measurements, excess arm volume, QuickDASH-9 scale	VR is beneficial in reducing postmastectomy lymphedema (*P*<.05) and can be used as an exercise-based technique in those who have undergone modified radical mastectomy with axillary lymph node dissection as it motivates and provides visual feedback to patients.
Buche et al [17], France	Pre–posttest	In a physiotherapy center, each of the 46 patients participated in 4 experimental conditions in a random order: 2 sessions used virtual immersion (ie, 1 participatory and 1 contemplative), 1 session proposed musical listening, and the fourth was a standard session care.	The Greener Gamer’s Nature Treks VR relaxation application has 9 relaxing visual environments with relaxing sounds, including 2 immersive modes: contemplative mode and participatory mode.	The sessions were performed over a period of 10 months in a physiotherapy center. Each session lasted an average 30 minutes.	ITC–SOPI^j^, feeling of elapsed time, SAI, QC^k^	An increase in positive emotions (ie, joy and happiness) and a decrease in anxiety regardless of which support methods were offered (*P*<.05). Participatory VR created a more intense feeling of spatial presence.
Jimenez et al [40], Australia	Quasi-experimental design study	Patients with breast cancer (n=18) in the control group received the standard pre-RT^l^ education package at a targeted cancer therapy center. Patients with breast cancer (n=19) in the experimental group attended a VERT^m^-based education session detailing RT immobilization, planning, and treatment.	The VERT education program incorporated low-level technical information about RT, patient anatomy, and radiation dose. Aspects of immobilization, simulation, planning, and treatment pertinent to patients with breast cancer were explored.	Each patient attended 1 session, with each session lasting 1 hour.	Radiation therapy knowledge and experience, STAI^n^	VERT breast cancer–targeted education programs are of high value, which can improve patients’ RT knowledge (*P*<.05) and decrease their anxiety (*P*>.05).
Bani et al [16], Jordan	RCT	Female patients with breast cancer (n=80) were randomly assigned to the intervention and comparison groups.	The intervention group chose 2 scenarios: deep sea diving “Ocean Rift” or sitting on the beach with the “Happy Place” track.	The VR exposure session was ended at the peak time of painkiller efficacy.	VAS, SAI, MMSE^o^	One session of immersive VR plus morphine resulted in a significant reduction in pain and anxiety self-reported scores, compared with morphine alone, in patients with breast cancer (*P*<.05).
House et al [30], USA	Pre–posttest	Community-dwelling women (n=6) with postsurgical breast cancer pain in the upper arm.	The BrightArm Duo Rehabilitation System consists of a low-friction robotic rehabilitation table, computerized forearm supports, a display, a laptop for the therapist station, a remote clinical server, and a library of custom integrative rehabilitation games.	The duration of the VR-based therapy sessions progressed from 20 to 50 minutes, twice a week for 8 weeks.	BDI-II^p^, BVMT-R^q^, TMT-A^r^, TMT-B^s^, NAB^t^, NPRS^u^, HVLT-R^v^, and PHQ-9^w^	Pain intensity showed a 20% downward trend. Outcomes indicate improvement in cognition, shoulder range, strength, function, and depression.
Schneider et al [15], USA	RCT: crossover design	A crossover design was used to examine the effects of a VR distraction intervention on chemotherapy-related symptom distress levels in 16 women aged ≥50 years.	Participants chose from 3 CD-ROM–based scenarios: Oceans Below, A World of Art, or Titanic: Adventure Out of Time.	Participants wore the head-mounted device during their intravenous chemotherapy treatment. Each scenario could last up to several hours.	MMSE, PFS^x^, SAI, SDS^y^	A significant decrease in the SAI (*P*=.10) scores was observed immediately following chemotherapy treatments when participants used VR. No significant changes were found in SDS or PFS values. There was a consistent trend toward improved symptoms on all measures 48 hours following completion of chemotherapy.
Schneider et al [41], USA	RCT: crossover design	A crossover design was used to examine the effects of a VR distraction intervention on chemotherapy-related symptom distress levels in 20 women aged 18-55 years.	Participants chose from 3 CD-ROM–based scenarios: deep sea diving, walking through an art museum, or solving a mystery.	During the chemotherapy infusions, participants received the VR distraction intervention for 45-90 minutes.	SDS, STAI, PFS, evaluation of VR intervention	The major findings of this study demonstrated that symptom distress and fatigue were significantly lower following chemotherapy treatment during which the VR intervention was implemented.
Jin et al [42], China	RCT	Patients with breast cancer (n=38) assigned to the experience group received VR-based training, and the other 38 patients with breast cancer in the control group received standard physical training.	A rehabilitation VR system including a video learning module, an action acquisition module, and an action scoring module.	A total of 3 months, 15–30 minutes per session, twice per day.	Adherence, ROM, the climbing height of finger, degree of edema.	The VR system with auxiliary game treatment was able to substantially improve limb function recovery, compliance, and subjective initiative in rehabilitation training, and reduce the edema of affected limbs (*P*<.05).
Zhu et al [43], China	RCT	Patients with breast cancer (n=80) who were randomly assigned to the experience group received VR-based training, while the control group received standard physical training.	Patients received VR-based shoulder and hand rehabilitation exercises.	A total of 3 months, 15–30 minutes per session, twice per day.	Adherence, ROM, the climbing height of finger, incidence of lymphedema	The VR rehabilitation system improved limb function recovery, compliance, and reduced the incidence of lymphedema (*P*<.05).
Chen et al [44], China	Pre–posttest	Patients with breast cancer (n=80) with cognitive impairment after chemotherapy.	The 80 patients received virtual cognitive intervention training.	An 8-week intervention	MoCA^z^, activities of daily living	The scores of the Montreal Cognitive Assessment Scale increased significantly and the scores of ADL were lower than those before the intervention (*P*<.05).

^a^RCT: randomized controlled trial.

^b^VR: virtual reality.

^c^VAS: visual analog scale.

^d^ROM: range of motion.

^e^DASH: disability of the arm, shoulder, and hand.

^f^TKS: Tampa Scale of Kinesiophobia.

^g^SAI: State Anxiety Inventory.

^h^SV-POMS: short version of Profile of Mood States.

^i^VRSQ: Virtual Reality Symptom Questionnaire.

^j^ITC–SOPI: Independent Television Commission–Sense of Presence Inventory.

^k^QC: a questionnaire on cybersickness.

^l^RT: radiation therapy.

^m^VERT: Virtual Environment for Radiotherapy Training.

^n^STAI: State-Trait Anxiety Inventory.

^o^MMSE: Mini-Mental State Examination.

^p^BDI-II: Beck Depression Inventory, Second Edition.

^q^BVMT-R: Brief Visuospatial Memory Test, Revised.

^r^TMT-A: Trail Making Test A.

^s^TMT-B: Trail Making Test B.

^t^NAB: Neuropsychological Assessment Battery.

^u^NPRS: Numeric Pain Rating Scale.

^v^HVLT-R: Hopkins Verbal Learning Test, Revised.

^w^PHQ-9: Patient Health Questionnaire.

^x^PFS: Piper Fatigue Scale.

^y^SDS: Symptom Distress Scale.

^z^MoCA: Montreal Cognitive Assessment.

### Assessment of the Risk of Bias of the Studies Included in the Review

**Figure 2:**
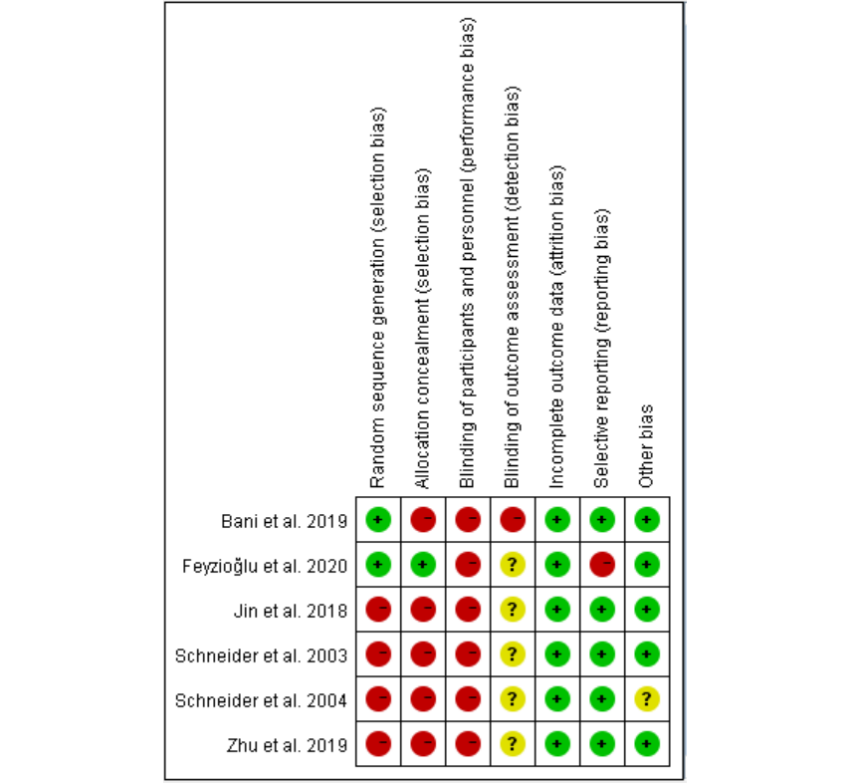
Risk of bias analysis of included randomized controlled trials.

**Figure 3:**
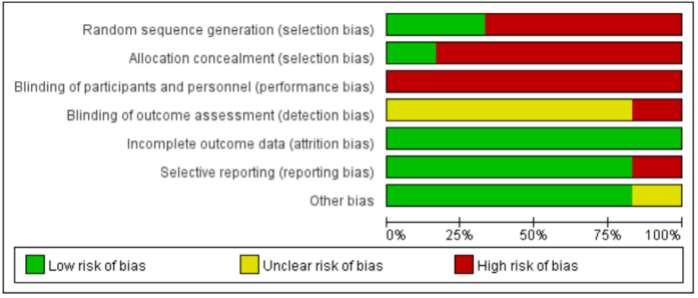
Overall risk of bias analysis of randomized controlled trials.

The results of the assessment of risk of bias are presented in Figures 2 and 3 and Table 3. The Cochrane risk of bias tool was used to assess the quality of the included RCTs. For RCTs, allocation concealment and blinding will not seriously influence the selection of patients and the measurement of outcomes. Two of the 6 RCTs reported randomized methods in detail [16,22], while the remaining 4 trials did not provide the methods of sequence generation, nor demonstrated that the participants were recruited randomly. None of the trials provided concealment methods, except 1 trial that reported the use of anonymization by placing numbers into opaque, sealed envelopes to conceal the allocation sequence [22]. In all trials, no blind method was used on participants due to the particularity of the intervention methods. None of the trials reported employing blinding of assessors, except for 1 trial that reported the person who collected the data [16]. Only 3 studies performed power calculations and reported adequate statistical power [16,22,41], while the other studies did not perform power calculations and were without dropouts [15,42,43].

ROBINS-I was used to assess the risk of non-RCTs. Four studies [14,17,40,44] had a moderate risk of bias, with confounding, outcome measurement, and selective reporting being the primary sources. Two studies [30,39] had a critical risk of bias due to missing data. The risk of selection bias was judged to be low for all studies. A detailed list of the risk of bias assessments is provided in Table 3.

**Table 3:** Overall risk of bias analysis of the nonrandomized controlled trials.

Study	Bias due to confounding	Selection bias	Bias in classification of interventions	Bias due to deviations from intended interventions	Bias due to missing data	Bias in measurement of outcomes	Bias in selection of the reported result	Overall bias
Chirico et al [14]	Moderate	Low	Low	Low	Low	Moderate	Moderate	Moderate
Atef et al [39]	Moderate	Low	Low	Moderate	Critical	Low	Low	Critical
Buche et al [17]	Moderate	Low	Low	Moderate	Moderate	Moderate	Moderate	Moderate
Jimenez et al [40]	Moderate	Low	Low	Moderate	Moderate	Moderate	Moderate	Moderate
House et al [30]	Moderate	Low	Low	Serious	Critical	Moderate	Moderate	Critical
Chen et al [44]	Moderate	Low	Low	Low	Low	Moderate	Moderate	Moderate

### Effects of Interventions

#### Shoulder Range of Motion

**Figure 4:**
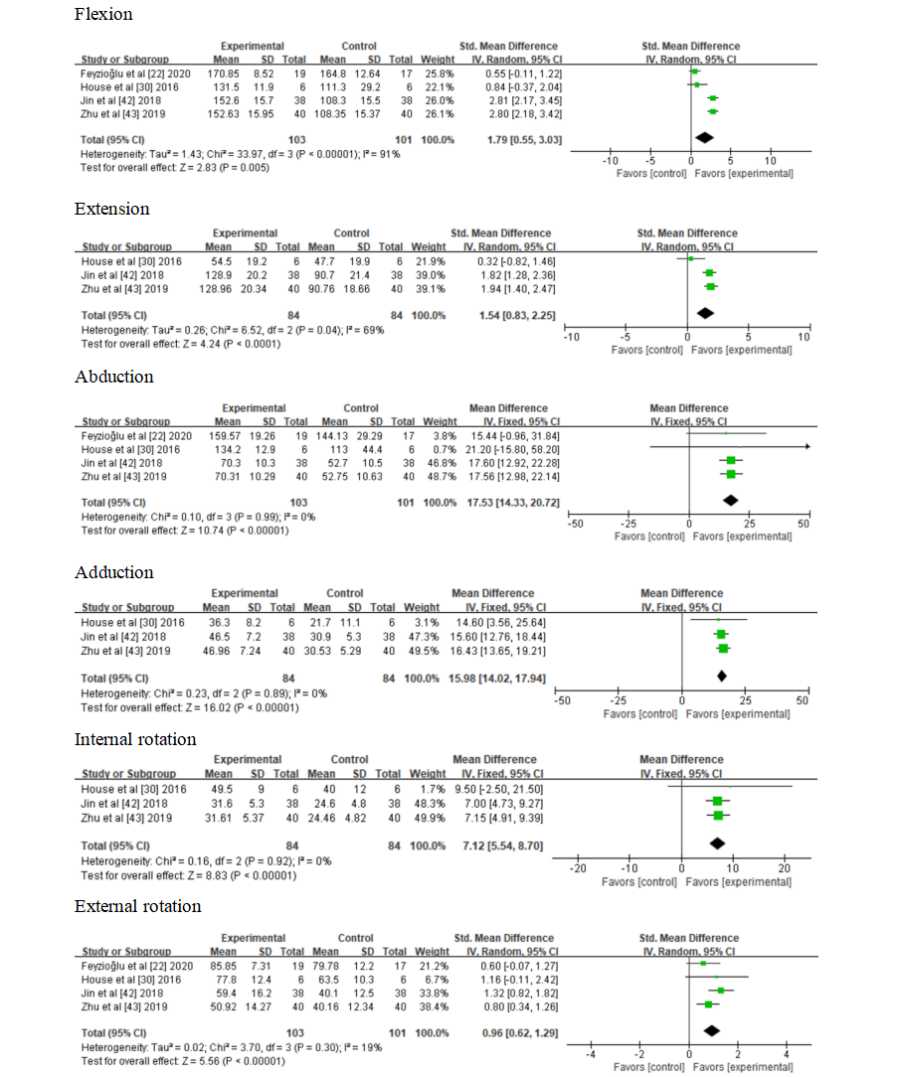
Forest plot assessing the effectiveness of using virtual reality-based interventions on shoulder range of motion.

A meta-analysis of 4 studies [22,30,42,43] suggested statistically significant results for VR-based interventions for upper shoulder ROM. ROM was measured in degrees using a digital goniometer. We observed that VR-based interventions were more effective than standard training, as shown in Figure 4. The statistical analysis showed significant results for flexion (standard mean difference [SMD] 1.79; 95% CI 0.55 to 3.03; *P*=.005), extension (SMD 1.54; 95% CI 0.83 to 2.25; *P*<.001), abduction (MD 17.53; 95% CI 14.33 to 20.72; *P*<.001), adduction (MD 15.98; 95% CI 14.02 to 17.94; *P*<.001), internal rotation (MD 7.12; 95% CI 5.54 to 8.70; *P*<.001), and external rotation (SMD 0.96; 95% CI 0.62 to 1.29; *P*<.001).

#### Hand Grip Strength

**Figure 5:**
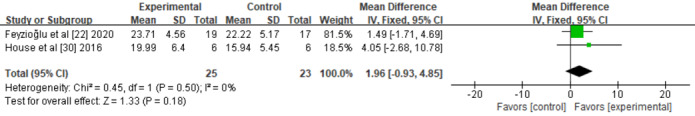
Forest plot assessing the effectiveness of using virtual reality-based interventions on hand grip strength.

Two studies [22,30] measured grip strength. The Saehan hydraulic hand dynamometer was used to measure grip strength. According to the *I*^2^ statistic, 0% of variation across studies was due to heterogeneity. This homogeneity was confirmed by the chi-square test (*P*=.50). A fixed-effect model was fitted. The study by House et al [30] reported better results than that by Feyzioğlu et al [22]. We observed that VR-based interventions were more effective than standard training. However, the overall result of this meta-analysis was not conclusive, as shown in Figure 5.

#### Anxiety

**Figure 6:**
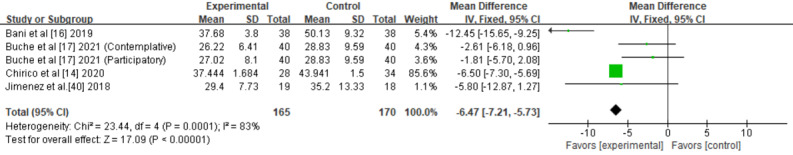
Forest plot assessing the effectiveness of using virtual reality-based interventions on anxiety.

Of the 10 studies, 4 assessed the influence of VR-based interventions on the severity of anxiety [14,16,17,40]. The severity of anxiety was measured using the State Anxiety Inventory [14,16,17] and the State-Trait Anxiety Inventory [40]. A meta-analysis of anxiety symptoms from 4 studies produced similar positive results favoring VR-based interventions over standard education, pharmacological interventions, or noninterventions. There was substantial heterogeneity (*P*<.0001; *I*^2^=83%), likely due to different duration, schedule, intensity, and type of interventions and methodological factors. Figure 6 shows the meta-analysis of the anxiety symptoms.

#### Depression

**Figure 7:**
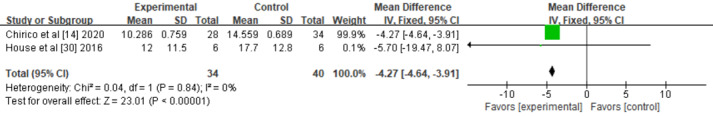
Forest plot assessing the effect of using virtual reality-based interventions on depression.

Of the 10 studies, 2 assessed the influence of VR-based interventions on the severity of depression [14,30]. The severity of depression was measured using the short version of Profile of Mood States and Beck Depression Inventory, Second Edition. A meta-analysis of depression from 2 studies produced similar positive results favoring VR-based interventions (MD −4.27; 95% CI −4.64 to −3.91; *P*<.001). Figure 7 shows the meta-analysis of the depression symptoms.

#### Pain

**Figure 8:**
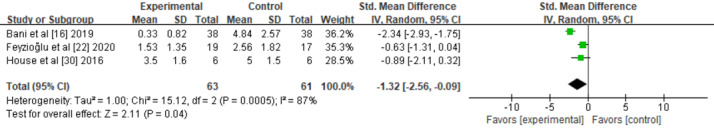
Forest plot assessing the effectiveness of using virtual reality-based interventions on pain.

Of the 10 studies, 3 assessed the influence of VR-based interventions on the severity of pain [16,22,30]. The severity of pain was measured using the visual analog scale. A meta-analysis of pain from 3 studies produced similar positive results favoring VR-based interventions over standard education, pharmacological interventions, or noninterventions. There was substantial heterogeneity (*P*<.001; *I*^2^=87%), likely due to different duration, schedule, intensity, and type of interventions and methodological factors. Figure 8 presents the meta-analysis of the pain symptoms.

#### Cognitive Function

**Figure 9:**
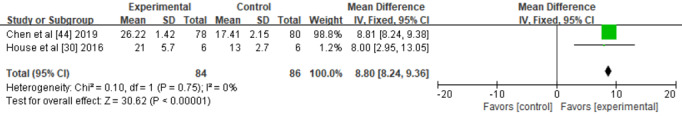
Forest plot assessing the effect of using virtual reality-based interventions on cognitive function (ie, verbal memory).

Of the 10 studies, 2 assessed the influence of VR-based interventions on cognition function [30,44]. Cognition function was measured using the Montreal Cognitive Assessment Scale and Brief Visuospatial Memory Test, Revised. A meta-analysis of cognitive function from 2 studies [30,44] produced similar positive results favoring VR-based interventions (MD 8.80; 95% CI 8.24 to 9.36; *P*<.001). Figure 9 presents the meta-analysis of the pain symptoms.

#### Fatigue

Chirico et al [14] reported significant changes in fatigue using the short version of the Profile of Mood States and revealed a significant difference between the 2 study groups (13.50 [SD 0.58] vs 15.03 [SD 0.53]; *P*<.001). Further studies are needed to explore the efficacy of VR-based rehabilitation interventions in reducing the level of fatigue.

#### Incidence of Complications

Of the 10 studies, 2 [42,43] focused on the incidence of complications after surgery. Jin et al [42] reported significant differences in lymphedema incidence between their 2 study groups (10.53% vs 42.11%; *P*<.05). Zhu et al [43] reported the incidence of total complications, such as lymphedema, bleeding, effusion, and flap necrosis, between their 2 study groups (12.50% vs 32.50%; *P*<.05).

#### Cybersickness Symptoms

Of the 10 studies, 4 [14,15,17,41] focused on cybersickness symptoms. Chirico et al [14**]** analyzed possible VR-associated cybersickness symptoms using the Virtual Reality Symptom Questionnaire. The findings showed that with the exception of a slight difficulty in concentrating, all symptoms (eg, headache, dizziness, nausea, eyestrain, drowsiness) occurred with a frequency less than 20%. Buche et al [17] used a questionnaire on cybersickness to evaluate the possible side effects (nausea, headache, and dizziness, etc.) of VR. The findings showed that 4 out of 46 patients (8.70%) experienced mild physical discomfort following VR. None of the patients in the other 2 studies [15,41] reported any unusual symptoms, such as dizziness, increased nausea, or visual disturbances.

#### Fear of Movement

Feyzioğlu et al [22] reported significant changes in fear of movement using the Tampa Kinesiophobia Scale and revealed a significant difference between the study 2 groups (29.47 [SD 5.31] vs 37.35 [SD 4.51]; *P*<.001). Further studies are needed to explore the efficacy of VR-based rehabilitation interventions in reducing the level of kinesiophobia.

## Discussion

### Principal Findings

This research aimed to use qualitative and quantitative methods to evaluate the effectiveness of VR-based interventions in the rehabilitation management of patients with breast cancer. Twelve studies were included in the systematic review, 10 of which were included in the meta-analysis. A total of 604 participants were involved in different studies. In view of our results, we can conclude that VR-based interventions are more effective in improving the emotional, cognitive, and physical well-being of BCSs. For other outcomes (and comparators), the evidence was less compelling in improving learners’ skills, attitudes, satisfaction, and patient-related outcomes.

### Quality of the Evidence

For RCTs, due to the nature of the intervention, we judged the majority of studies to be at a high risk of bias for nonblinding of participants and study personnel and nonblinding of outcome assessment. Although blinding of outcome measurement would not seriously influence the results, nonblinding of participants might bias the effect [45]. For non-RCTs, most of the studies had a moderate risk of bias in confounding, outcome measurement, and selective reporting. Some studies had a serious or critical risk of bias, most frequently due to the outcome measurement, missing data, and choice of analyses, which did not allow controlling for missing data.

Moreover, the lack of randomization and power analysis to calculate the appropriate sample size, as well as different duration, schedule, intensity, and type of interventions, and different scales of measurements all contributed to the heterogeneity of the included studies. Additionally, some outcomes could not be analyzed quantitatively due to data format (eg, incidence of complications, cybersickness symptoms) or the fact that the data were only reported in 1 study (eg, fatigue, fear of movement). Furthermore, we could not assess the risk of publication bias because funnel plot–based methods are not accurate for less than 10 included studies per outcome.

### Overall Completeness and Applicability of the Evidence

We identified 12 studies; however, most of them were limited by small sample sizes. Therefore, additional studies are needed to confirm our findings. We are encouraged that a number of larger RCTs are currently underway [46-52], which are likely to inform the field further.

Our findings must be viewed with caution owing to the limited number of trials with low quality. Moreover, as we only included a small number of trials, it was not feasible to conduct subanalyses regarding VR-based interventions or study design. A more empirical study is needed to determine the applicability of VR-based interventions in BCSs according to intense physical and psychological symptoms, function defects, and adverse effects. Additionally, further study is needed to standardize the contents of VR-based interventions, especially for upper limb recovery. Moreover, to successfully implement VR-based rehabilitation exercises into daily practice, it is better to provide detailed information on training frequency, duration of the intervention, and targeted motor skills [53].

### Potential Biases in the Review Process

Although we performed extensive searches of the literature, there is a possibility that we did not identify all relevant studies. Two review authors independently completed data screening, extraction, evaluation of risk of bias, and certainty of evidence rating. Any discrepancies between the reviewers were resolved by discussion or in consultation with other reviewers in the event that disagreement persisted. Even though we contacted all relevant study authors for additional information, we did not always receive a response. Low study quality, inadequate methodological details, and significant inconsistencies across trials decrease the overall quality of the evidence. Moreover, the variability in VR-based interventions, as well as the timing, regimens, and definitions of outcome measurements all have the potential to contribute to inaccuracies in the assessment of the intervention effects.

### Agreement With Other Studies or Reviews

The findings of this systematic review and meta-analysis indicated that VR-based interventions have a positive effect on physical and psychological symptom management and ROM. Our findings are a valuable extension of recently published systematic reviews and meta-analyses. Previous similar studies mainly focused on the effect of immersive or nonimmersive VR-based interventions in cancer survivors or stroke survivors; however, the results of the studies were inconsistent.

Ahmad et al [54] and Chow et al [55] reported that VR-based interventions may be more effective in the management of pain and anxiety in patients with cancer, whereas a nonsignificant difference in pain and anxiety was reported by Zeng et al [56]. By contrast, Ioannou et al [57] reported that VR-based interventions demonstrated a trend toward improvement in pain. One possible explanation for this difference is that the intervention effects of immersive and nonimmersive systems differ and that these reviews focused on cancer survivors. Moreover, different cancer survivors may have various physical and psychological symptoms that lead to variability within the findings. Therefore, it is necessary to conduct controlled trials between different interventions and populations. In addition, although no significant improvement was observed in most studies, we cannot ignore the potential health-promoting effects of VR-based interventions. In agreement with our review, Aminov et al [58] found evidence of a significant effect in improving upper limb function using VR-based rehabilitation interventions and suggest VR as an adjunct for stroke rehabilitation.

Overall, previous reviews have presented similar conclusions to those of our review, suggesting that although the evidence is limited, it does exist. However, the results of this review should be interpreted with caution due to the limited number of controlled trials analyzed, the small sample sizes, and low methodological quality. The majority of previous reviews/meta-analyses indicate that more high certainty of evidence is needed before VR-based interventions can be considered as potential strategies for rehabilitation management in BCSs consistently.

### Implications for Research and Practice

The examination of VR-based interventions is recommended to ascertain whether there is a role for technology‐based exercise in improving the late and long-term side effects of breast cancer treatment. Furthermore, empirical evidence is required to provide well‐substantiated recommendations regarding the frequency, duration, and content of the VR intervention. Finally, future studies on VR-based interventions could utilize more consistent reference standards, such as standardizing the frequency, duration, and content of the VR interventions. Such standardization minimizes bias and heterogeneity between studies. Future studies could focus on (1) the late and long-term side effects of breast cancer management; (2) the mechanism of symptom management; and (3) combination of VR with artificial intelligence, physiological indexes, and electroencephalogram.

### Conclusions

The late and long-term side effects resulting from breast cancer treatment are persistent and prominent. The findings from this review suggest that VR has the potential to facilitate immediate and longer-term improvements in symptom management and the performance of upper extremity function following a surgery for BCSs. Although the use of VR-based interventions has expanded in the rehabilitation management of BCSs, the current evidence for using VR-based interventions for both immediate and long-term improvements among BCSs remains limited. Future trials would benefit from using multicenter data, with larger sample sizes, longer follow-up periods, and high methodological quality.

## Appendix

Multimedia Appendix 1 Search Strategy.

## Abbreviations

BCRL: breast cancer–related lymphedema
BCS: breast cancer survivor
MD: mean difference
PICOS: Population, Intervention, Comparison, Outcomes and Study
PRISMA: Preferred Reporting Items for Systematic Reviews and Meta-Analyses
PROSPERO: Prospective Register of Systematic Reviews
RCT: randomized controlled trial
ROBINS-I: Risk Of Bias In Non-randomized Studies of Interventions
ROM: range of motion
SMD: standard mean difference
VR: virtual reality
