# Supplementary material for: The Effectiveness of Virtual Reality–Based Interventions in Rehabilitation Management of Breast Cancer Survivors: Systematic Review and Meta-analysis
Source: JMIR Serious Games. 2022 Feb 28;10(1):e31395. doi: 10.2196/31395 (PMC8922144; doi:10.2196/31395)
Supplement: Multimedia Appendix 1 [file games_v10i1e31395_app1.doc]

**2021.5.25**

Web of science176+ embase385+ pubmed147+ cochrane39+ CINHAL69+Wangfang 40 + CNKI 74+VIP 7+sinomed 27=964

**Web of science**

| #3 | 176 | #2 AND #1 |
| --- | --- | --- |
| #2 | 746,578 | TS=("Breast Neoplasms"[Mesh] OR “breast neoplasms” OR "breast neoplasm" OR ”breast tumors” OR “breast tumor” OR “breast cancer” OR “mammary cancer” OR “mammary cancers” OR “Breast Malignant Neoplasm” OR “Breast Malignant Neoplasms” OR “Malignant Tumor of Breast” OR “Breast Malignant Tumor” OR “Breast Malignant Tumors” OR “Cancer of Breast” OR “Cancer of the Breast” OR “Mammary Carcinoma” OR “Human Mammary Carcinomas” OR “Human Mammary Carcinoma” OR “Mammary Neoplasms” OR “Human Mammary Neoplasm” OR “Human Mammary Neoplasms” OR “Mammary Neoplasm” OR “Breast Carcinoma” OR “Breast Carcinomas”) |
| #1 | 90,628 | TS=("Virtual Reality"[Mesh] OR VR OR "virtual reality" OR "virtual environment" OR "head-mounted display" OR "virtual reality goggle") |

**Embase**

| #3 | 385 | #1 AND #2 |
| --- | --- | --- |
| #2 | 640261 | 'breast neoplasms' OR 'breast neoplasm' OR 'breast tumors' OR 'breast tumor' OR 'breast cancer' OR 'mammary cancer' OR 'mammary cancers' OR 'breast malignant neoplasm' OR 'breast malignant neoplasms' OR 'malignant tumor of breast' OR 'breast malignant tumor' OR 'breast malignant tumors' OR 'cancer of breast' OR 'cancer of the breast' OR 'mammary carcinoma' OR 'human mammary carcinomas' OR 'human mammary carcinoma' OR 'mammary neoplasms' OR 'human mammary neoplasm' OR 'human mammary neoplasms' OR 'mammary neoplasm' OR 'breast carcinoma' OR 'breast carcinomas' |
| #1 | 66857 | 'virtual reality' OR vr OR 'virtual environment' OR 'head-mounted display' OR 'virtual reality goggle' |

**Pubmed**

| #3 | 147 | #1 AND #2  Search: #1 AND #2  ("Virtual Reality"[MeSH Terms] OR ("proc ieee virtual real conf"[Journal] OR "vr"[All Fields]) OR "Virtual Reality"[All Fields] OR "virtual environment"[All Fields] OR "head-mounted display"[All Fields] OR "virtual reality goggle"[All Fields]) AND ("Breast Neoplasms"[MeSH Terms] OR "Breast Neoplasms"[All Fields] OR "breast neoplasm"[All Fields] OR "breast tumors"[All Fields] OR "breast tumor"[All Fields] OR "breast cancer"[All Fields] OR "mammary cancer"[All Fields] OR "mammary cancers"[All Fields] OR "Breast Malignant Neoplasm"[All Fields] OR "Breast Malignant Neoplasms"[All Fields] OR "Malignant Tumor of Breast"[All Fields] OR "Breast Malignant Tumor"[All Fields] OR "Breast Malignant Tumors"[All Fields] OR "Cancer of Breast"[All Fields] OR "Cancer of the Breast"[All Fields] OR "Mammary Carcinoma"[All Fields] OR "Human Mammary Carcinomas"[All Fields] OR "Human Mammary Carcinoma"[All Fields] OR "Mammary Neoplasms"[All Fields] OR ("Breast Neoplasms"[MeSH Terms] OR ("breast"[All Fields] AND "neoplasms"[All Fields]) OR "Breast Neoplasms"[All Fields] OR ("human"[All Fields] AND "mammary"[All Fields] AND "neoplasm"[All Fields])) OR "Human Mammary Neoplasms"[All Fields] OR "Mammary Neoplasm"[All Fields] OR "Breast Carcinoma"[All Fields] OR "Breast Carcinomas"[All Fields]) |
| --- | --- | --- |
| #2 | 44085 | #2"Breast Neoplasms"[MeSH Terms] OR "Breast Neoplasms"[All Fields] OR "breast neoplasm"[All Fields] OR "breast tumors"[All Fields] OR "breast tumor"[All Fields] OR "breast cancer"[All Fields] OR "mammary cancer"[All Fields] OR "mammary cancers"[All Fields] OR "Breast Malignant Neoplasm"[All Fields] OR "Breast Malignant Neoplasms"[All Fields] OR "Malignant Tumor of Breast"[All Fields] OR "Breast Malignant Tumor"[All Fields] OR "Breast Malignant Tumors"[All Fields] OR "Cancer of Breast"[All Fields] OR "Cancer of the Breast"[All Fields] OR "Mammary Carcinoma"[All Fields] OR "Human Mammary Carcinomas"[All Fields] OR "Human Mammary Carcinoma"[All Fields] OR "Mammary Neoplasms"[All Fields] OR ("Breast Neoplasms"[MeSH Terms] OR ("breast"[All Fields] AND "neoplasms"[All Fields]) OR "Breast Neoplasms"[All Fields] OR ("human"[All Fields] AND "mammary"[All Fields] AND "neoplasm"[All Fields])) OR "Human Mammary Neoplasms"[All Fields] OR "Mammary Neoplasm"[All Fields] OR "Breast Carcinoma"[All Fields] OR "Breast Carcinomas"[All Fields] |
| #1 | 23984 | #1"Virtual Reality"[MeSH Terms] OR "proc ieee virtual real conf"[Journal] OR "vr"[All Fields] OR "Virtual Reality"[All Fields] OR "virtual environment"[All Fields] OR "head-mounted display"[All Fields] OR "virtual reality goggle"[All Fields] |

Cochrane

(("Virtual Reality" OR VR OR “virtual reality” OR “virtual environment” OR “head-mounted display” OR "virtual reality goggle")):ti,ab,kw AND (("Breast Neoplasms" OR “breast neoplasms” OR "breast neoplasm" OR ”breast tumors” OR “breast tumor” OR “breast cancer” OR “mammary cancer” OR “mammary cancers” OR “Breast Malignant Neoplasm” OR “Breast Malignant Neoplasms” OR “Malignant Tumor of Breast” OR “Breast Malignant Tumor” OR “Breast Malignant Tumors” OR “Cancer of Breast” OR “Cancer of the Breast” OR “Mammary Carcinoma” OR “Human Mammary Carcinomas” OR “Human Mammary Carcinoma” OR “Mammary Neoplasms” OR “Human Mammary Neoplasm” OR “Human Mammary Neoplasms” OR “Mammary Neoplasm” OR “Breast Carcinoma” OR “Breast Carcinomas”)):ti,ab,kw"

CINAHL for full text

| 序号 | 检索表达式 |
| --- | --- |
| S3 | S1 AND S2 |
| S2 | "Breast Neoplasms" OR “breast neoplasms” OR "breast neoplasm" OR ”breast tumors” OR “breast tumor” OR “breast cancer” OR “mammary cancer” OR “mammary cancers” OR “Breast Malignant Neoplasm” OR “Breast Malignant Neoplasms” OR “Malignant Tumor of Breast” OR “Breast Malignant Tumor” OR “Breast Malignant Tumors” OR “Cancer of Breast” OR “Cancer of the Breast” OR “Mammary Carcinoma” OR “Human Mammary Carcinomas” OR “Human Mammary Carcinoma” OR “Mammary Neoplasms” OR “Human Mammary Neoplasm” OR “Human Mammary Neoplasms” OR “Mammary Neoplasm” OR “Breast Carcinoma” OR “Breast Carcinomas” |
| S1 | "Virtual Reality"[Mesh] OR VR OR “virtual reality” OR “virtual environment” OR “head-mounted display” OR "virtual reality goggle" |

Sinomed

| 序号 | 检索表达式 | 结果 |
| --- | --- | --- |
| 3 | (#2) AND (#1) | 27 |
| 2 | 乳腺癌 | 119912 |
| 1 | 虚拟现实 OR 虚拟现实技术 OR VR OR VR技术 OR 3D眼镜 OR VR眼镜 OR 头盔显示器 OR 头戴式显示器 | 7144 |

VIP

U=( 乳腺癌 OR 乳腺肿瘤 OR 乳腺瘤 OR 乳腺癌症 OR 乳房肿瘤) AND U=(虚拟现实 OR 虚拟现实技术 OR VR OR VR技术 OR 3D眼镜 OR VR眼镜 OR 头盔显示器 OR头戴式显示设备)

Wangfang

主题:(乳腺癌+乳腺肿瘤+乳腺瘤+乳腺癌症+乳房肿瘤)*主题:(虚拟现实+虚拟现实技术+VR+VR技术+3D眼镜+VR眼镜+头盔显示器+头戴式显示设备)

CNKI

SU= '乳腺癌'+ '乳腺肿瘤'+'乳腺瘤'+'乳腺癌症'+ '乳房肿瘤' AND SU = '虚拟现实'+ '虚拟现实技术'+ 'VR'+ 'VR技术'+ '3D眼镜'+ 'VR眼镜'+ '头盔显示器'+ '头戴式显示设备'
